# Intersensory redundancy impedes face recognition in 12-month-old infants

**DOI:** 10.3389/fpsyg.2023.1210132

**Published:** 2023-07-17

**Authors:** Aslı Bursalıoğlu, Alexandria Michalak, Maggie W. Guy

**Affiliations:** Department of Psychology, Loyola University Chicago, Chicago, IL, United States

**Keywords:** infancy, intersensory redundancy, face recognition, visual attention, look duration

## Abstract

This study examined the role of intersensory redundancy on 12-month-old infants’ attention to and processing of face stimuli. Two experiments were conducted. In Experiment 1, 72 12-month-olds were tested using an online platform called Lookit. Infants were familiarized with two videos of an actor reciting a children’s story presented simultaneously. A soundtrack either matched one of the videos (experimental condition) or neither of the videos (control condition). Visual-paired comparison (VPC) trials were completed to measure looking preferences for the faces presented synchronously and asynchronously during familiarization and for novel faces. Neither group displayed looking preferences during the VPC trials. It is possible that the complexity of the familiarization phase made the modality-specific face properties (i.e., facial characteristics and configuration) difficult to process. In Experiment 2, 56 12-month-old infants were familiarized with the video of only one actor presented either synchronously or asynchronously with the soundtrack. Following familiarization, participants completed a VPC procedure including the familiar face and a novel face. Results from Experiment 2 showed that infants in the synchronous condition paid more attention during familiarization than infants in the asynchronous condition. Infants in the asynchronous condition demonstrated recognition of the familiar face. These findings suggest that the competing face stimuli in the Experiment 1 were too complex for the facial characteristics to be processed. The procedure in Experiment 2 led to increased processing of the face in the asynchronous presentation. These results indicate that intersensory redundancy in the presentation of synchronous audiovisual faces is very salient, discouraging the processing of modality-specific visual properties. This research contributes to the understanding of face processing in multimodal contexts, which have been understudied, although a great deal of naturalistic face exposure occurs multimodally.

## Introduction

Face perception plays an important role in social communication and understanding of the world around us. Facial stimuli are very salient, which is evident from the first days of life (Goren et al., 1975; Valenza et al., 1996; Turati et al., 2002). In daily life, faces are typically perceived in a multimodal and dynamic way. For example, when a person speaks, we can see their face, its movement, and hear their voice at the same time. This multimodal presentation is characterized by intersensory redundancy (IR), which refers to the synchronous presentation of information across senses (i.e., the auditory information we hear is in synchrony with the visual information we see) (Bahrick and Lickliter, 2000).

Bahrick and Lickliter (2000) proposed an intersensory redundancy hypothesis that describes how IR guides infant attention. There are three assumptions to the IR hypothesis. First, IR attracts infants’ attention, especially to amodal stimulus properties. Amodal properties are properties that are not specific to one sensory modality and can be sensed across multiple modalities (e.g., rhythm can be perceived visually when viewing a bouncing ball and can be heard as well). Amodal information is very salient for infants when it is presented with IR, compared to nonredundant information. Second, when multimodal information is presented redundantly, the redundant information will be processed and learned earlier than non-redundant information. When a property of an event is synchronously presented in more than one modality, it will be detected earlier, learned, and remembered prior to other properties. Third, perceptual and processing advantages for amodal information will help infants to perceive multimodal events with IR as unitary. For example, if they see a bouncing ball and hear it tapping the surface in temporal synchrony, they will perceive the sight and sound as belonging together. Additionally, Bahrick et al. (2004) predicted that the detection and processing of modality-specific properties, which are properties that can only be perceived through one sensory modality (e.g., color, pitch), will be more salient and easier to process in unimodal contexts. Essentially, there will be an advantage for processing amodal stimulus properties when stimuli are presented with IR, but there will be an advantage for processing modality-specific stimulus properties when IR is not present. Definitions of the major concepts are included in Table 1.

**Table 1:** Definition of major concepts.

Term	Definition
Intersensory redundancy	The synchronous presentation of information across different sensory modalities
Amodal properties	Properties that are not specific to one sensory modality (e.g., can be perceived both auditorily and visually)
Modality-specific properties	Properties that can only be perceived by a single sensory modality (e.g., only perceived by vision)
Unimodal stimulation	Stimulation that pertains to only one sensory modality
Multimodal stimulation	Stimulation that pertains to two or more sensory modalities

In a foundational study, Bahrick and Lickliter (2000) tested 5-month-olds’ perception of rhythm, an amodal property, across three experiments. Infants were habituated to multimodal synchronous, multimodal asynchronous, visual unimodal, and auditory unimodal events involving a hammer tapping in a specific rhythm. Infants’ visual recovery in response to a new rhythm was assessed in order to understand if they recognized the new rhythm. The results showed that infants could identify changes in the rhythm only when they were habituated to the multimodal synchronous presentation. When habituated to the multimodal asynchronous and unimodal presentations, they did not discriminate between the familiar and novel rhythmic patterns. These results indicate that IR greatly contributes to infants’ ability to attend to, perceive, and learn amodal properties.

In the presence of IR, amodal properties are salient and are processed and learned early (e.g., Bahrick et al., 2019), which comes at the expense of modality-specific properties. Alternatively, under unimodal conditions, modality-specific properties (e.g., facial configuration) are perceived and learned earlier than amodal properties. This means that the processing of modality-specific stimuli, such as faces, may be hindered when they are perceived in multimodal conditions, such as when seeing someone speak (e.g., Bahrick et al., 2013, 2014; Hillairet de Boisferon et al., 2021). For example, in a series of experiments, 2- and 3-month-olds were shown synchronous audiovisual, asynchronous audiovisual, and unimodal visual presentations of faces (Bahrick et al., 2013). The results showed that 2-month-olds could not detect novel faces following synchronous audiovisual familiarization, but they were able to do so following asynchronous audiovisual and unimodal familiarization. The synchrony did not impact face recognition in 3-month-olds. Similarly, Hillairet de Boisferon et al. (2021) found that 9- and 12-month-old infants were able to recognize a face paired with their native language, but not with a non-native language, however, this was only evident when the native or non-native soundtrack was matched with a static picture of a face. When the soundtrack was paired with a dynamic face, neither age group showed recognition of the faces. These may show that dynamic audiovisual familiarization attracts attention to amodal properties, directing it away from modality-specific properties and inhibiting face recognition. These findings are in line with the IR hypothesis, which suggests that IR guides infants’ attention to the amodal properties of events and may hinder the processing of modality-specific visual properties, which would be relevant for face recognition.

Much of the research investigating the role of IR on infant attention has been conducted with 5-month-old infants (e.g., Bahrick and Lickliter, 2000; Bahrick et al., 2010; Kraebel and Armstrong, 2012; Reynolds et al., 2014). However, Bahrick et al. (2004) predicted that older infants, who have more experience perceiving and interpreting the outside world, would more easily identify amodal and modality-specific information in unimodal and multimodal contexts. Although emerging face specialization is seen around the end of the first year of life (e.g., Halit et al., 2003; Conte et al., 2020), less is known about how IR might interact with specialization in face processing around 12 months of age. Face detection during early infancy is driven by a subcortical pathway (Simion et al., 1998; Johnson, 2005), which is shown to be sensitive to face configuration and facilitates orientation to faces in infants (Morton and Johnson, 1991). This subcortical pathway preferentially responds to faces and promotes face exposure, which is critical for the development of cortical regions associated with face specialization. As infants get older and gain experience with faces, this heightened face exposure leads to the cortical areas becoming more specialized for faces (Morton and Johnson, 1991). For example, Conte et al. (2020) used event-related potentials (ERPs) to study neural responses to static faces and objects in 3- to 12-month-olds. They examined the N290, which is an ERP component associated with the development of face specialization. They found that the N290 was larger in amplitude in response to faces than objects only at 9 and 12 months of age, indicating that face processing responses become more specialized across the first year of life.

### Present study

In the current study, we examined the effects of intersensory redundancy on face recognition at 12 months of age. Morton and Johnson (1991) proposed that a shift from subcortical to cortical face processing occurs over the first 2 months of life, and neural studies show increased face specialization around 12 months of age, while younger infants demonstrate more immature processing of faces (e.g., Halit et al., 2003; Guy et al., 2016; Conte et al., 2020; Chen et al., 2021). Twelve-month-olds were selected because they are expected to be attracted to the amodal properties of the faces presented synchronously (Lewkowicz and Hansen-Tift, 2012; Pons et al., 2015), while also being able to process the unimodal aspects of the stimuli (Bahrick and Lickliter, 2004; Bahrick et al., 2006). While most existing literature on face processing and recognition is based on static, unimodal stimuli, there has been a recent push to increase the representation of dynamic stimuli (e.g., Ujiie et al., 2020; Hillairet de Boisferon et al., 2021; Nakagawa et al., 2023). In this study we used dynamic, audiovisual stimuli to investigate the role of IR in face processing. Dynamic stimuli may provide insight into more naturalistic face processing, as faces are typically interacted with in multimodal settings. Additionally, by presenting infants with dynamic stimuli, it is possible to understand the effects of intersensory redundancy on attention to and recognition of faces. Beyond 4 months of age, infants have been shown to demonstrate greater attention to synchronous, multimodal faces than silent faces, synchronous, multimodal objects, or silent objects (Bahrick et al., 2016).

In this study, we tested face recognition using familiarization and visual-paired comparison trials (VPCs). Familiarization is a commonly used behavioral method when studying infant attention and cognition (e.g., Shultz and Bale, 2001; Clearfield and Westfahl, 2006; Karmiloff-Smith et al., 2010; Poulin-Dubois et al., 2022). During a familiarization trial, infants are presented with a stimulus of interest and their looking time is recorded. It is expected that after sufficient exposure to the stimulus, infants’ interest and looking time will diminish and that they will become familiarized to the stimulus (Aslin, 2007). The familiarization trial is commonly followed by a visual-paired comparison (VPC) task, which can be used to assess visual attention and recognition memory (Fagan, 1990). During this procedure, the infants are shown pairs of stimuli for a set amount of time. If they look longer to one than the other, it implies that they can discriminate between the two stimuli and prefer one. Infants are naturally attracted to novel stimuli (Pascalis and de Schonen, 1994) and VPCs are often used to test if a novelty preference exists. Additionally, if the processing of a stimulus is incomplete, infants often display a familiarity preference (Houston-Price and Nakai, 2004). A familiarity preference is characterized by looking longer to the stimulus the infant has just been familiarized with during the VPC procedure and is believed to reflect an early phase of processing (Roder et al., 2000). A familiarity preference may also be observed when there is limited exposure during familiarization (e.g., Rose et al., 1982; Richards, 1997), or when there is a longer delay between familiarization and test trials (e.g., Bahrick and Pickens, 1995; Bahrick et al., 1997; Courage and Howe, 1998). The VPC procedure has been established to be a reliable measure of recognition memory in infancy in humans (e.g., Pascalis et al., 2002; Quinn et al., 2020) as well as non-human primates (e.g., Nemanic et al., 2004; Sliwa et al., 2011). This procedure does not require any verbal response, which makes it appropriate for using with infants.

We used Lookit, an online data collection platform for developmental researchers (Scott et al., 2017; Scott and Schulz, 2017), to complete the current study. Through Lookit, researchers can create studies, recruit and contact families, and collect study data. Families who have internet and webcam access can use this platform to participate in studies. Studies are not conducted in real time, so families participate on demand at their convenience. Using Lookit helps to recruit more diverse samples and its data quality allows for in-depth analysis of looking behavior (Nelson and Oakes, 2021). Lookit has been utilized in diverse recent studies examining infant development (e.g., Nelson and Oakes, 2021; Luchkina and Waxman, 2022; Rocha and Addyman, 2022; Wang, 2022; Colomer and Woodward, 2023).

For this study, we recruited two groups of 12-month-old infants. In Experiment 1, infants were familiarized with two videos that were presented side-by-side simultaneously, the videos depicted two actors speaking. In the experimental condition, the audio was synchronous with one actor’s video, while in the control condition, it did not match either video. This meant that as the infants watched the actors recite stories, the audio stimulus they heard was from another story. In Experiment 2, infants were familiarized with one video of an actor speaking. In the synchronous condition, the audio was synchronous with the video, and in the asynchronous condition, it was asynchronous with the video. Hypotheses for Experiments 1 and 2 are presented in Table 2 and described in the text.

**Table 2:** Study hypotheses.

**Experiment 1**
(1) Infants in the **experimental condition** will demonstrate *attraction to the synchronous familiarization stimulus. Processing of amodal stimulus properties will be prioritized* and will interfere with processing of the modality-specific face characteristics. *Recognition of the synchronous face is not expected* during the VPC trials, but infants may show recognition of the asynchronous face.
(2) Infants in the **control condition** *will not show a preference for either of the two asynchronous faces during familiarization*, resulting in equivalent processing of familiar faces.
(3) There will be a *relationship between looking times to asynchronous stimuli during familiarization and VPC trials*. Increased looking to an asynchronously presented face during familiarization will be negatively correlated with looking to the same face during the VPC trials.
**Experiment 2**
(1) Infants familiarized with a **synchronous face** will be attracted to the amodal rather than the modality-specific properties of the face, leading to less skilled processing and recognition. These participants *will not show recognition of the familiar face* during the VPC.
(2) Infants familiarized with an **asynchronous face** will allocate their attention to the modality-specific properties of a face that are relevant for recognition *and will recognize the familiarized face* during the VPCs.

## Experiment 1

### Introduction

In Experiment 1, 12-month-old infants were familiarized with videos of two faces presented simultaneously, where the audio matched only one of the faces (i.e., experimental condition) or it matched neither face (i.e., control condition). Infants were presented with visual paired comparison (VPC) trials following familiarization. These trials included the presentation of the familiar faces together, as well as each familiar face paired with a novel face. Looking preferences were measured to investigate infants’ face recognition.

We had multiple hypotheses for Experiment 1. First, infants in the experimental condition were expected to demonstrate sensitivity to synchrony and attraction to the synchronous familiarization stimulus (e.g., Bahrick et al., 2016, 2018; Curtindale et al., 2019), indicated by increased looking to the synchronous face during familiarization. Because the amodal stimulus properties are most salient, we expected that infants would be prevented from attending to modality-specific face properties, discouraging recognition of the synchronous familiar face during the VPC trials. If infants demonstrated stimulus recognition, it was expected to be seen for the asynchronous familiar face, which was presented without IR, allowing for modality-specific processing. Second, infants in the control condition were not expected to show a preference for either of the two asynchronous faces, resulting in equivalent processing of the familiar faces. Modality-specific face properties may be processed because neither familiar stimulus possesses IR. This would be reflected in a looking preference for novel over familiar faces during the VPC trials. Third, we expected to observe a relationship between looking times to asynchronous stimuli during familiarization and VPC trials. Specifically, we expected that increased looking to an asynchronously-presented face during familiarization would be negatively correlated with looking to the same face during the VPC trials. We did not expect to see this pattern for the experimental group, as the IR would draw infants’ attention to amodal stimulus properties, interfering with modality-specific face properties. Study hypotheses for Experiment 1 and Experiment 2 are presented in Table 2.

### Methods and materials

#### Participants

Seventy-two 12-month-olds (age *M* = 358.85 days, *SD* = 15.37, 34 females, 1 non-binary) participated in Experiment 1. Additional participants were recruited, but their data were removed due to the parent facing the screen (*N* = 1), the infant’s eyes not being visible in the recordings (*N* = 1), incomplete recordings (*N* = 6), and technical issues (*N* = 5). To be eligible for participation, infants had to be born full-term (i.e., 38 weeks), and have regular exposure to English as reported by their parents. In addition to English, 26 participants were exposed to at least one other language. Additional demographic information for Experiment 1 is included in Table 3.

**Table 3:** Demographic information for study participants.

Participant categories	Experiment 1		Experiment 2	
	Experimental group	Control group	Synchronous group	Asynchronous group
Age in days	359.29	357.94	355.91	360.61
Gender				
Female	21	12	20	13
Male	19	19	14	10
Nonbinary	1	0	0	0
Race				
American Indian or Alaska Native	0	1	0	0
Asian	4	3	1	0
Black or African American	1	0	0	2
Caucasian or White	27	21	29	19
Mixed	7	5	4	2
Ethnicity				
Hispanic or Latino	3	3	3	3
Not Hispanic or Latino	32	25	27	15
Language				
English monolingual	30	16	25	17
Bilingual or multilingual	11	15	9	6

We recruited participants through Lookit (Scott et al., 2017; Scott and Schulz, 2017). Studies actively collecting data are advertised on Lookit’s homepage and the families who are on the Lookit database receive an email when their child is eligible for a study. Additionally, families in the Loyola University Center for Research in Child Development database were invited to participate in this study *via* email. We also invited families to participate in this study through posts on our social media pages (i.e., Facebook and Instagram) and social media advertisements. Families were compensated with $5 Amazon e-gift cards to thank them for their time. Prior to participation in the study, the families provided their verbal consent by recording a video, as approved by the Institutional Review Board at Loyola University Chicago. Families were able to participate in the study on demand, as the study was not controlled in real time by a live researcher.

#### Stimuli and apparatus

##### Familiarization stimuli

**Figure 1:**
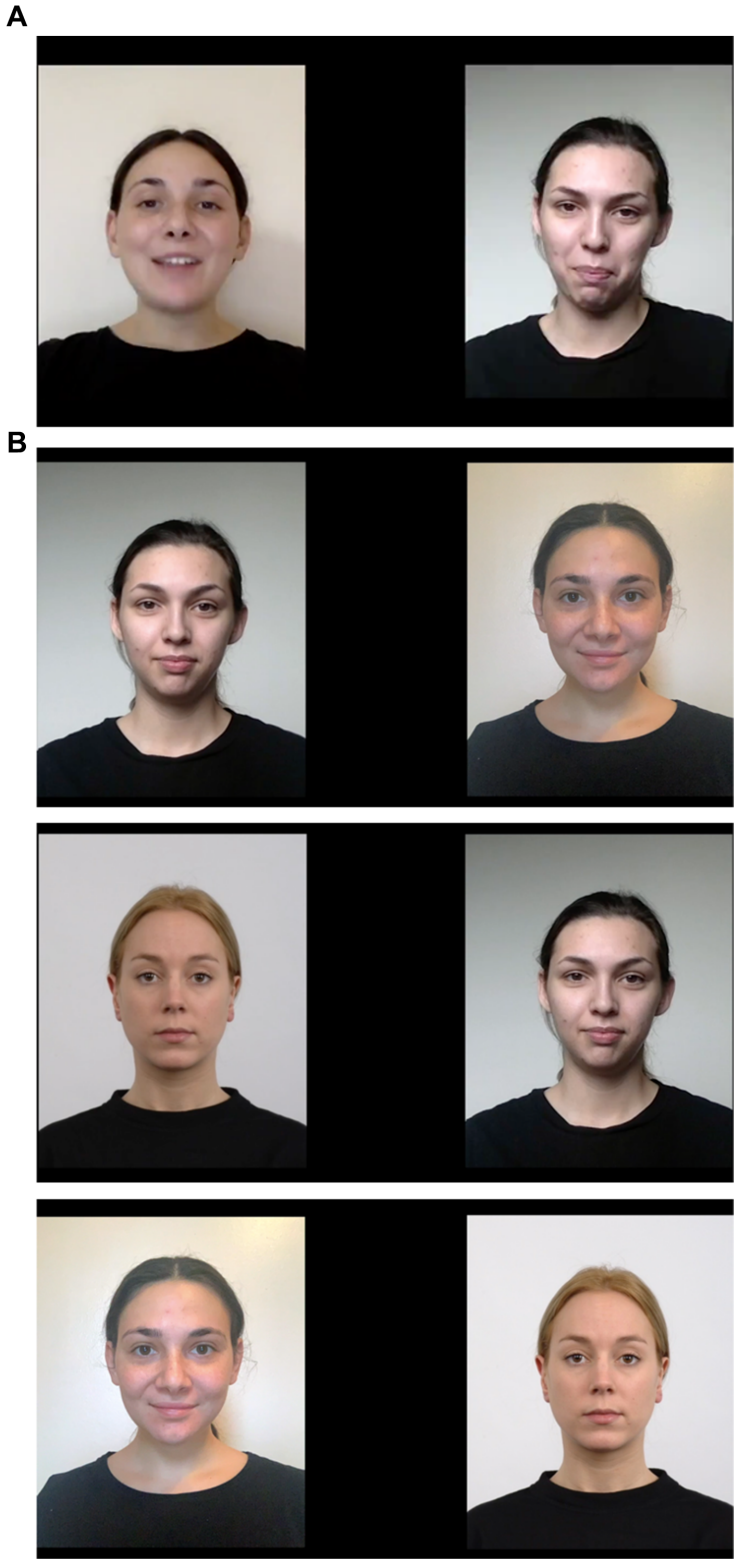
**(A)** In experimental condition, one of the faces was presented synchronously with the audio component. For the control condition, neither face was synchronous with the audio. **(B)** Familiarized faces were paired together with each other and with a novel stimulus to assess looking preferences. The stimuli were switched after every few participants.

During the familiarization phase, infants were presented with two side-by-side videos simultaneously. In these videos, two White, female actors recited children’s stories in an infant-directed manner. The videos of the actors reciting children’s stories were presented with an audio recording of a children’s story that was synchronous with one or neither of the two videos. Synchrony was observed when the movement of the actor’s face and mouth temporally matched the story that was heard auditorily (i.e., the actor was visually and auditorily reciting the same children’s story in a temporally synchronous manner). Asynchrony was shown when temporal synchrony was not observed (i.e., the actor was visually reciting a different children’s story than what was heard auditorily so temporal synchrony was not achieved). In the experimental condition, auditory synchrony was achieved with one video presented, but not the other. In the control condition, the audiovisual components of the stimuli were asynchronous because the auditory stimulation heard did not temporally match either video presented. The actors stood against a light neutral background, wore black shirts, had no jewelry or makeup on, and tied their hair back. Only their faces and shoulders were visible. A sample familiarization stimulus is presented in Figure 1A. The familiarization phase lasted for 30 s, as several past studies had familiarization phase lasting for 30 s (e.g., Jusczyk and Aslin, 1995; Trainor et al., 2004) or were designed to have infants acquire 20 s of looking during familiarization (e.g., Reynolds and Richards, 2005; Bornstein and Mash, 2010; Reynolds et al., 2011; Guy et al., 2013, 2017). Rose (1983) also reported that the mean exposure time required to accumulate 20 s of looking time was 28.3 s.

##### Visual paired comparison stimuli

During VPC trials, infants viewed pairs of static photographs of female faces that were presented side-by-side. These included the faces that were viewed during familiarization, as well as a novel White female face. The faces had a neutral expression, and the actors were against a light neutral background, wearing black shirts with no jewelry or makeup, and their hair tied back. Example VPC stimuli are presented in Figure 1B. Each paired comparison lasted for 7.5 s. Stimulus selection was randomized so that all participants did not see the same actors in the videos and photographs that they viewed.

##### Apparatus

In order to participate in this study via Lookit, the parent or caregiver had to have Internet access and use Google Chrome or Firefox browsers. They also had to use a desktop or a laptop computer and have a working webcam, speaker, and microphone.

#### Procedure

**Figure 2:**
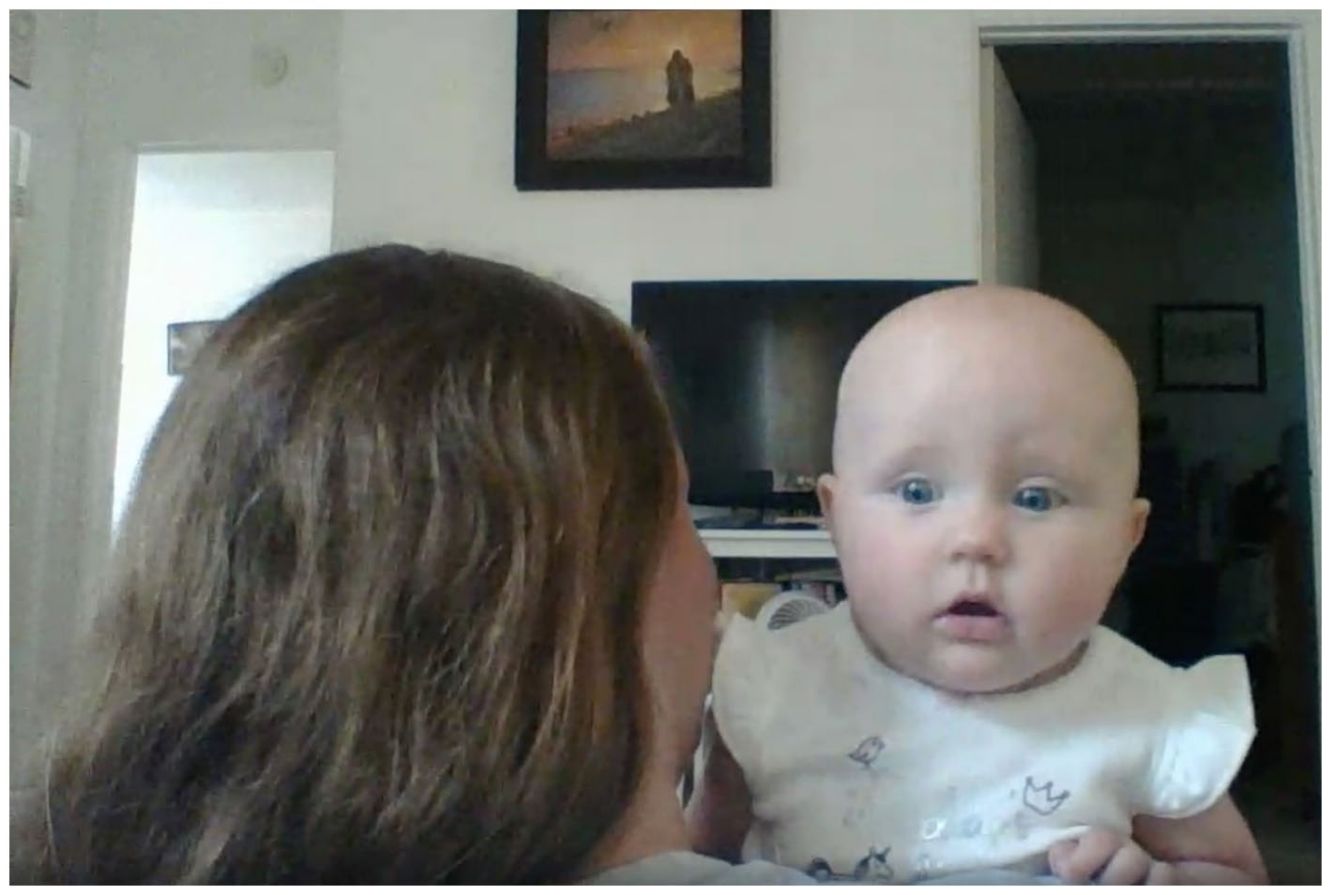
Participant getting into position. All parents/legal guardians were asked to sit facing back the computer monitor.

The parent or legal guardian provided verbal consent and filled out a demographic form prior to the start of data collection. They were given the option to watch a 10 s preview of the familiarization video. After that, they were asked to position themselves to have their back face the screen and have their child look at the screen over their shoulder. Figure 2 shows a participant and their parent as they get into position. A calibration video was presented and the familiarization commenced, which lasted for 30 s for both experimental and control conditions. The participants in the experimental condition viewed two side-by-side videos with the soundtrack matching one video, whereas participants in the control condition viewed two side-by-side videos that did not match the soundtrack. Familiarization was followed by three VPC trials, including three pairs of pictures: (1) the two faces that they viewed during familiarization, (2) one of the familiar faces paired with a novel face, and (3) the other familiar face paired with a novel face. Each pair was on the screen for 7.5 s. The order of the VPC presentations and position of the faces (i.e., right or left) was randomized across participants. At the end of the experiment, parents were allowed to select whether their data would be used for private (i.e., viewed only by authorized scientists including the research team), scientific and educational (i.e., videos shared for scientific and educational purposes), or public (i.e., videos share on the Lookit website and news articles about the study) purposes. At this point, they were also given the option to withdraw their data if they preferred.

#### Coding

The data were coded using the Datavyu software (Datavyu Team, 2014). For each participant, four videos were coded: one familiarization video and three VPC videos. Videos were coded for infants’ gaze direction to the right side of the screen, left side of the screen, or off-screen. As the study was completed without a researcher present in real time, we had less control over the testing environment, which led us to adopt strict criteria for looking times. To be included in further analyses, infants had to spend at least 20 s looking at the screen during the 30 s familiarization (e.g., Rose et al., 1982; Colombo et al., 2001; Courage et al., 2006), and 5 s looking at the screen during the 7.5 s VPC trials (e.g., Reynolds et al., 2010). Infants’ eyes had to be visible and the parent had to be facing away from the screen for their data to be included in the final sample. Additionally, we used calibration stimuli prior to the presentation of the familiarization videos. Before we coded each participants’ video, we viewed the calibration to see if the infant was responding to different attractors, in a way that allowed us to discriminate right and left gaze. This helped to ensure that the participants were not in a distracting environment. We also utilized this data to ensure that participants did not display a side bias.

#### Statistical analyses

Analyses were conducted using IBM SPSS. For the test trials, looking time was calculated as the ratio of looking time towards the novel stimulus relative to the accumulated looking time to novel and familiar stimuli. Our dependent variables included looking time, measured in seconds, as well as the proportion of looking to one face over the other one during the VPC trials. These measures allowed us to investigate looking preferences during familiarization and VPC trials. Multiple analytical strategies were employed to examine differences in looking behavior within and across groups, including (1) one-sample t-tests to test for novelty preferences by determining if look durations to the novel stimulus were above the chance value of 50% (e.g., Yamashita et al., 2011; Wagner et al., 2020), and (2) correlations to examine whether individual differences in participants’ patterns of looking during familiarization were associated with their looking behavior during the VPCs. Our approach in this study included simultaneous presentation of two dynamic stimuli during familiarization. We specifically wanted to see if longer looking to the asynchronous face during familiarization would lead to shorter looking to the same face during the VPC trials.

### Results

In the experimental group, there were 40 12-month-olds. In the control group, there were 32 12-month-olds. Table 4 shows the mean total looking times and standard deviations during familiarization and VPC trials for each group of participants.

**Table 4:** Mean of total looking times for familiarization and VPCs(s).

	Experimental group		Control group	
	*M*	SD	*M*	SD
Familiarization	28.75	2.93	29.37	2.69
VPC – two familiar faces	7.05	0.65	7.25	0.58
VPC^a^ – familiar vs. novel	7.07	0.67	7.07	0.66
VPC^b^ – familiar vs. novel	6.84	0.81	7.12	0.74

^a^Async-fam vs. novel face for the experimental group, async-left-fam vs. novel face for the control group. ^b^Sync-fam vs. novel face for the experimental group, async-right-fam vs. novel face for the control group.

#### Analysis 1: looking time during familiarization

Looking time to each of the familiar stimuli were measured and one-sample t-tests were computed to determine if the percentage of look duration to either face was above the chance value of 50% for participants in the experimental and control conditions. Infants in the experimental condition demonstrated a mean looking time of 28.75 s. They spent 45.40% of their time looking at the synchronous face (*M* = 13.05 s), and 54.60% of their time looking at the asynchronous face (*M* = 15.70 s). This difference reaches marginal statistical significance, *t*(39) = −1.856, *p* = 0.071, indicating a stronger tendency to look at the asynchronous face during familiarization in the experimental condition. For the control group, the mean total looking time was 29.37 s; infants spent 46.97% of their time looking to the asynchronous face displayed on the left side of the screen (*M* = 13.85 s) and 53.03% of their time looking to the face displayed on the right side of the screen (*M* = 15.52 s). This difference was not significant, *t*(31) = 1.279, *p* = 0.210, showing that infants in the control condition did not prefer one face over the other during familiarization.

#### Analysis 2: looking time during VPCs

**Figure 3:**
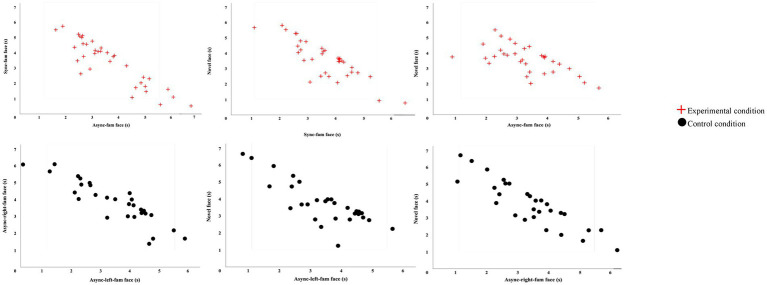
Looking times during the visual paired-comparison trials(s). Figure displays looking times during each visual paired-comparison trail for experimental and control conditions.

Data collected during the VPCs were used to test for looking preferences, indicative of recognition of the familiar stimulus. One-sample *t*-tests were run for each condition to test for novelty preference against a chance value of 50%. In the experimental condition, VPCs included: (1) the synchronous-familiar (sync-fam) face and the asynchronous-familiar (async-fam) face, (2) the async-fam face and the novel face, and (3) the fam-sync face and the novel face. In the control condition, VPCs included: (1) the asynchronous familiar face that had been viewed on the right side of the screen (async-right-fam) and the asynchronous familiar face that had been viewed on the left side of the screen (async-left-fam), (2) the async-left-fam face and the novel face, and (3) the async-right-fam face and the novel face. Figure 3 displays participants’ looking time during the VPC trials.

##### Experimental group

The total looking time during the VPC trials is presented in Table 4. It can be seen that total looking time is similar across all trials. When looking was examined based on stimulus type during each trial, we found that participants looked longer to the async-fam face (*M* = 3.69 s) than the sync-fam face (*M* = 3.36 s). They also looked longer to the novel face (*M* = 3.55 s) than the async-fam face (*M* = 3.29 s) and to the novel face (*M* = 3.54 s) than the sync-fam face (*M* = 3.53 s).

One-sample t-test analyses were conducted to test for looking preferences significantly different from the chance value of 50%. The results showed that they spent on average 52.67% of their time looking at the async-fam face versus the sync-fam face, *t*(34) = 0.350, *p* = 0.840, 52.31% of their time looking at the novel face versus the async-fam face, *t*(32) = 1.052, *p* = 0.301, and 50.14% of their time looking at the sync-fam face versus the novel face, *t*(34) = 0.051, *p* = 0.959. Thus, 12-month-olds in the experimental group did not show any significant novelty or familiarity preferences during the VPC trials.

##### Control group

Twelve-month-old in the control condition showed no significant differences in their looking times toward faces presented during the VPC trials. Total mean looking times for the VPC trials are presented in Table 4. Within the VPC trials, participants looked longer at the right-async-fam (*M* = 3.83 s) compared to the left-async-fam face (*M* = 3.42 s), the novel (*M* = 3.74 s) compared to the left-async-fam face (*M* = 3.38 s), and the novel face (*M* = 3.78 s) compared to the right-async-fam face (*M* = 3.29 s). One sample t-test showed that they spent on average 53.02% of their time looking at the right-async-fam face versus the left-async-fam face, *t*(29) = 0.927, *p* = 0.362, 52.27% of their time looking at the novel face versus the left-async-fam face, *t*(28) = 0.769, *p* = 0.448, and 53.45% of their time looking at the novel face versus the right-async-fam face, *t*(28) = 1.012, *p* = 0.320, showing no novelty or familiarity preferences during the VPC trials.

#### Analysis 3: correlations between looking patterns during familiarization and visual paired comparisons

Pearson product–moment correlations were run for each experimental condition to understand the relationship between looking time during familiarization and looking preferences during VPC trials. For the experimental group, looking time to the sync-fam face during familiarization was not significantly correlated with looking time to the same face during the VPC with two familiar faces (*r* = 0.104, *N* = 35, *p* = 0.553). Looking time to the async-fam face during familiarization was not significantly correlated with looking time to that face during the VPC trial with two familiar faces (*r* = −0.007, *N* = 35, *p* = 0.968). In the control group, the relationship between looking time to the right-async-fam face during familiarization and looking time to the same face during the VPC trial with two familiar faces was not significant (*r* = −0.228, *N* = 30, *p* = 0.226). The relationship between looking time to the left-async-fam face during familiarization and the VPC trial also did not reach significance (*r* = 0.052, *N* = 30, *p* = 0.787).

### Discussion

In Experiment 1, we investigated the processing of pairs of faces presented simultaneously, with or without intersensory redundancy. Specifically, we looked at how synchronous and asynchronous presentation of speaking faces affect attention to and recognition of faces in 12-month-old infants. The results showed that 12-month-olds did not have novelty or familiarity preferences during the VPCs. However, some evidence indicated that participants in the experimental condition processed the asynchronous familiar face more thoroughly than the synchronous familiar face. In the experimental condition, participants looked longer to the asynchronous face than the synchronous face during familiarization and looked longer to the novel face than the async-fam face when they were presented together in a VPC trial, although these differences did not reach statistical significance. The lack of significance may indicate that not all participants demonstrated this pattern of looking or that the async-fam face was not fully processed. Similarly, in the control condition, participants looked longer to the novel face compared to the asynchronous faces they had already viewed during familiarization. Once again, these effects did not reach significance, but could indicate that exposure to the asynchronous faces during familiarization resulted in some processing of that face.

**Figure 4:**
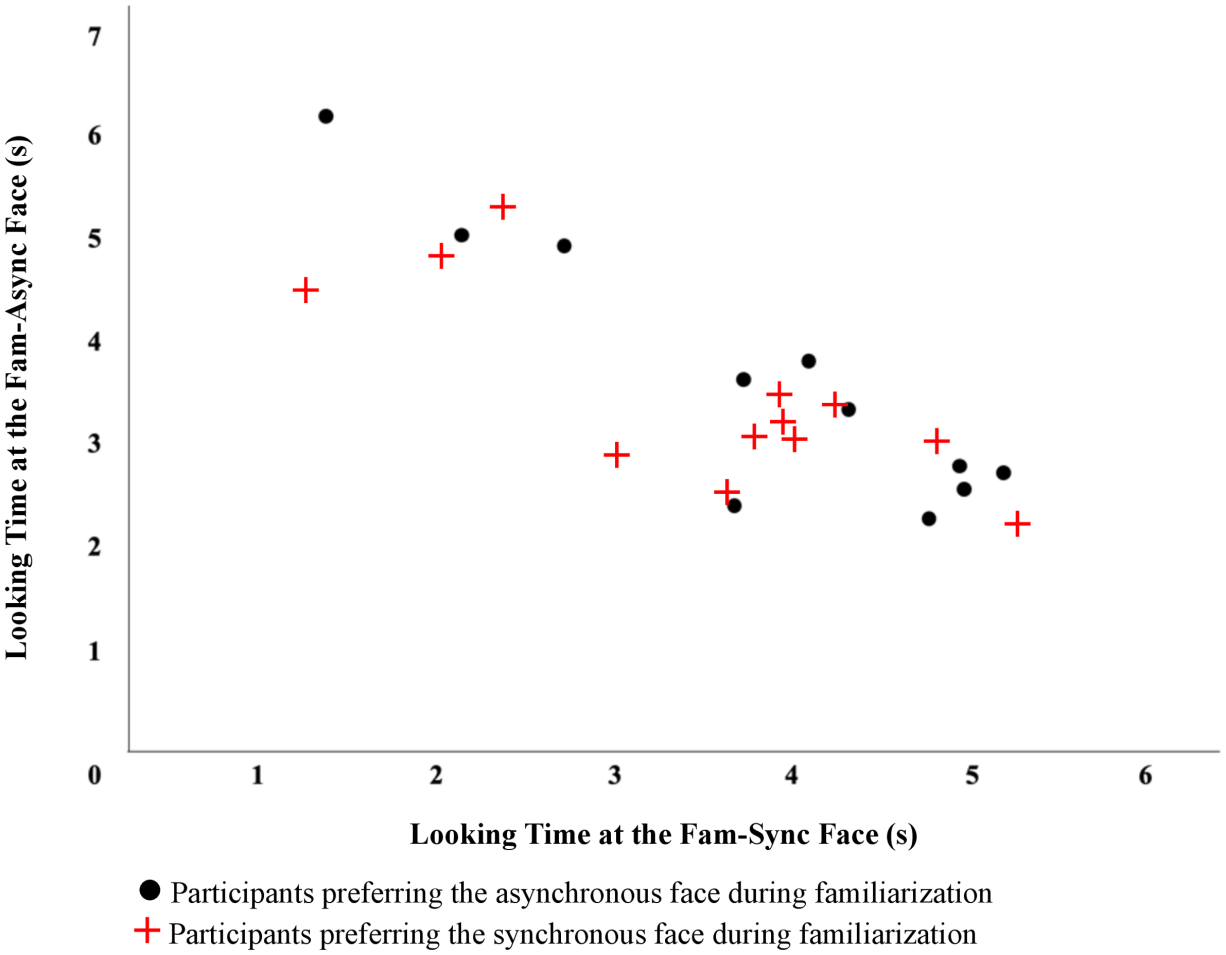
Familiar-synchronous vs. familiar-asynchronous looking times for the visual paired-comparison(s). Looking patterns of participants in the experimental condition that had a preference towards the synchronous or asynchronous face during the visual paired-comparison are plotted in the figure.

To better understand these non-significant trends and based on the observation of individual differences in looking behavior during familiarization, we divided the participants in the experimental condition into three groups based on amount of time looking to each of the familiar faces. We found that the third of participants that spent the longest amount of time looking to the async-fam face during familiarization (*N* = 13) also spent more time looking to the sync-fam face relative to the async-fam face when they were paired together in the VPC trial (see Figure 4). This may indicate that these infants processed some modality-specific properties of the asynchronous face during familiarization and recognized the asynchronous face during the VPC trial. Because the presence of IR for the synchronous face might have distracted them from the modality-specific properties of the synchronous face that would be necessary for face recognition, they did not show recognition of this face and treated it as more novel (compared to the asynchronous face). When we examined the third of participants in the experimental group who looked longest to the sync-familiar face during familiarization (*N* = 13), we did not see a similar pattern. Most of them continued to look longer to the sync-fam face compared to the async-fam face when they were paired in the VPC trial. This indicates that during familiarization, they may not have been able to move their attention beyond the amodal property (i.e., speech) and process the modality-specific properties of the face.

We also divided the participants in the control condition into three groups based on amount of time looking to each of the familiar faces. We found that the third of participants that spent the longest amount of time looking to the left asynchronous face during familiarization (*N* = 11) also spent more time looking to the right-async-fam face relative to the left-async-fam face when they were paired together in the VPC trial. Similarly, the third of the participants that spent the longest time looking to the right-async-fam (*N* = 11) during familiarization spent more time looking at the left-async-fam face compared to the right-async-fam face when they were paired together.

Presentation of complex, multimodal information can lead infants’ attention to amodal properties of the stimulus, while driving attention away from the components relevant for recognizing faces. Hillairet de Boisferon et al. (2021) recently found that when 9- and 12-month-old infants were familiarized with dynamic, speaking faces, they showed no evidence of face discrimination based on native language. However, when they were familiarized with static pictures of faces paired with a soundtrack containing speech of their native language, they were better able to discriminate the familiar face from a novel face. Their results show that even towards the end of the first year of life, presence of intersensory redundancy may guide infants’ attention away from the properties related to face recognition.

The familiarization procedure used in the current study was more complex (i.e., two dynamic faces presented simultaneously) than previous studies. It is possible that infants did not display a novelty preference because of the complexity of the familiarization procedure, which may have impeded a shift in attention to the modality-specific features of the face, facilitating recognition. In Experiment 2, we familiarized infants with one dynamic face presented with or without IR to explore whether 12-month-old infants can move beyond amodal stimulus properties to modality-specific stimulus properties.

## Experiment 2

### Introduction

In Experiment 2, 12-month-old infants were familiarized with one audiovisual video of a speaking actor to investigate whether they could move their attention beyond the amodal properties and direct their attention to modality-specific information within a multimodally presented face. This video was presented either synchronously (i.e., synchronous condition) or asynchronously (i.e., asynchronous condition). First, we hypothesized that participants familiarized with a synchronous audiovisual face would be attracted to the amodal rather than the modality-specific properties of the face, leading to less skilled processing and recognition of that face compared to participants who are familiarized with the asynchronous audiovisual face (e.g., Bahrick et al., 2013). We did not expect to see recognition of the synchronous-familiar face during the VPCs. Second, participants who were familiarized with an asynchronous audiovisual face were expected to allocate their attention to the modality-specific properties of a face that are relevant for recognition, as the asynchrony should not be salient or distracting. We expected this to be indicated by a novelty preference during the VPCs. Overall, this task was expected to facilitate greater recognition than Experiment 1 because participants only viewed one face during familiarization.

### Materials and methods

#### Participants

Fifty-six 12-month-olds (age *M* = 354.48 days, *SD* = 17.22, 21 females) participated in Experiment 2. The eligibility criteria were the same as in Experiment 1. In Experiment 2, nine participants were exposed to at least one other language than English. Additional demographic information is presented in Table 3. The distribution of the participants in terms of average age and gender across experimental groups was similar to Experiment 1 (see Table 3 for details). Recruitment strategies and compensation were the same as in Experiment 1.

#### Stimuli and apparatus

##### Familiarization stimuli

Familiarization was identical to Experiment 1, except that instead of side-by-side videos, infants were presented with a single 30 s video during familiarization (i.e., one group viewed the video with a synchronous audio, while the other group viewed the video with an asynchronous audio).

##### VPC stimuli

The VPC stimuli included three pairs of faces: (1) familiar face A paired with novel face B, (2) familiar face A paired with novel face C, (3) novel face B paired with novel face C. The number of VPC trials were modeled to be similar to Experiment 1. This also helped us to identify any side or face preferences that was not related to the stimuli or hypotheses. For the analysis, we used the first VPC that met looking criteria.

##### Apparatus

The apparatus was identical to Experiment 1.

#### Procedure

The procedure was identical to Experiment 1. The only exception was the familiarization procedure, which included one synchronous or asynchronous video for Experiment 2.

#### Coding

Coding was identical to Experiment 1, except that familiarization videos, were coded as looking to the stimulus or away from the stimulus, instead of indicating gaze direction to left or right sides of the monitor.

#### Statistical analyses

Analyses were conducted using IBM SPSS. For the test trials, looking time was calculated as the ratio of looking time towards the novel stimulus to the accumulated looking time to novel and familiar stimuli. Multiple analytical strategies were employed to examine differences in looking behavior within and across groups, including (1) one-sample t-tests to test for novelty preferences by determining if look durations to the novel stimulus were above the chance value of 50% (e.g., Yamashita et al., 2011; Wagner et al., 2020) and (2) correlations to examine whether individual differences in participants’ patterns of looking during familiarization were associated with their looking behavior during the VPCs.

### Results

In the synchronous group, there were 33 12-month-olds. In the asynchronous group, there were 23 12-month-olds. During the period of recruitment for the asynchronous group, we had fewer total enrollments compared to the synchronous group. We ran power analyses to find the power to be 0.745, which indicated that our current sample size is sufficient. Visual inspection of the data revealed that one participant demonstrated an atypical pattern of looking in one direction; we identified this participant as an outlier and ran further analyses without them. Table 5 shows the mean total looking times and standard deviations during familiarization and VPC for the synchronous and asynchronous groups.

**Table 5:** Mean of total looking times for familiarization and visual paired-comparison(s).

	Synchronous group		Asynchronous group	
	*M*	SD	*M*	SD
Familiarization	28.22	2.89	26.01	3.14
VPC – familiar vs. novel	7.10	0.71	6.87	0.69

#### Analysis 1: looking time during familiarization

Looking time to each of the familiar stimuli were measured and independent sample t-tests were computed to determine if the look duration of synchronous and asynchronous groups were significantly different than one another during familiarization. Infants in the synchronous condition demonstrated a mean looking time of 28.22 s. For the asynchronous group, the mean total looking time was 26.01 s. Further analyses showed that this difference between looking times of the groups were significant, *t*(54) = −2.708, *p* = 0.009, indicating that infants in the synchronous condition looked longer during familiarization than infants in the asynchronous condition.

#### Analysis 2: looking time during VPCs

**Figure 5:**
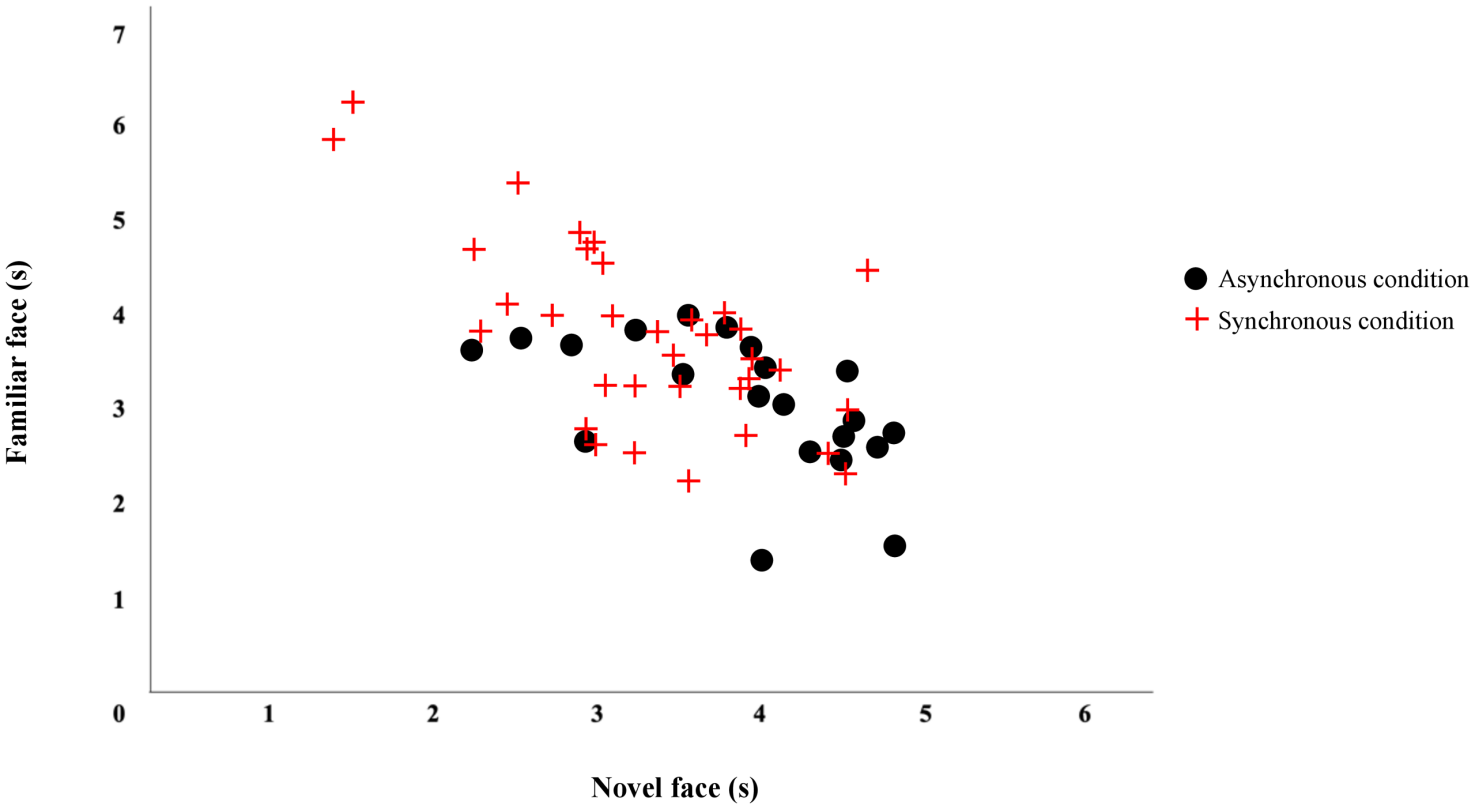
Looking time during the visual paired-comparison(s). The figure displays looking time during the VPC with the familiar and novel faces.

Data collected during the VPCs were used to test for novelty preferences, indicative of recognition of the familiar stimulus. The number of VPC trials were modeled to be similar to Experiment 1. One-sample t-tests were run for each group to test for novelty preference against a chance value of 50%. In both groups, VPCs included the familiar face and a novel face. Looking patterns for both groups are displayed in Figure 5.

##### Synchronous group

The total looking time for participants during the VPC trial was 7.10 s. One sample t-test analyses were conducted to identify if looking preferences of participants were significantly different from the chance value of 50%. The results demonstrated that they spent on average 3.79 s looking at the familiar face and 3.31 s looking at the novel face during the VPC. They did not show any significant novelty or familiarity preferences during the VPC trial, *t*(32) = −1.695, *p* = 0.100.

##### Asynchronous group

The total looking time during the VPC was 6.87 s. We conducted a one-sample t-test to examine if participants looked at either face significantly different than the chance value of 50%. We found that on average, they looked at the familiar face for 3.06 s and the novel face for 3.81 s. They looked at the novel face for 55% of the total looking time, which was different than the chance value, *t*(20) = 2.541, *p* = 0.019. This indicates that infants in the asynchronous condition demonstrated a novelty preference on the VPC trials.

Additionally, we compared the looking times between groups during the VPCs. There was a significant difference for the looking time to the familiar face between the synchronous and asynchronous groups. On average, participants in the synchronous group looked longer to the familiar face during the VPC (3.79 s) compared to the participants in the asynchronous group (3.06 s), *t*(52) = −3.092, *p* = 0.003). Average looking time to the novel face was also significantly different between the synchronous (3.31 s) and asynchronous groups (3.81 s), *t*(52) = 2.411, *p* = 0.019.

#### Analysis 3: correlations between looking patterns during familiarization and visual paired-comparison trials

##### Synchronous group

Looking time during familiarization was positively and significantly correlated with looking time to the sync-fam face (*r* = 0.434, *N* = 33, *p* < 0.05) in the VPCs.

##### Asynchronous group

Correlation analyses between look times during familiarization and VPC trials yielded no significant results (*r* = 0.086, *N* = 21, *p* = 0.712).

### Discussion

In Experiment 2, we found that 12-month-old infants paid more attention to synchronous than asynchronous multimodal stimuli, which indicates that temporal synchrony influences attention allocation at 12 months. Although participants paid more attention to the synchronous stimulus compared to the asynchronous stimulus, as indicated by longer looking time, they did not show any signs of recognition of the synchronous stimulus during the VPC trial. This indicates that stimulus synchrony recruited attention to amodal stimulus properties, and discouraged the processing of modality-specific properties, such as features related to face recognition.

Additionally, we found that infants in the asynchronous condition displayed a novelty preference during the VPC, which is indicative of stimulus recognition. This shows that one multimodal information is not presented in synchrony (i.e., when IR is not present), it is easier to process modality-specific information, supporting the IRH. Faces presented without IR were more thoroughly processed, which contributed to stimulus recognition, as evidenced by a novelty preference, on the VPC trial.

## General discussion

In this study, we investigated the effect of IR on face processing in 12-month-old infants. Specifically, we examined how synchronous and asynchronous presentation of speaking faces affect attention to and recognition of faces. We expected that when audiovisual faces were presented synchronously, or with IR, they would recruit infants’ attention. Because IR is very salient, we expected infants’ attention to be drawn to the amodal stimulus properties, and predicted that they would not be able to focus on the modality-specific properties of the faces that are necessary for recognition. In Experiment 1, we tested this by familiarizing infants with two side-by-side videos of a woman speaking, where the soundtrack was synchronous with only one video or neither video. The results indicated that infants did not recognize the familiar stimuli on the VPC trials, whether they had been presented synchronously or asynchronously. However, infants that paid more attention to the asynchronous face during the familiarization phase were more likely to look longer at the synchronous familiar face (versus the asynchronous familiar face) during the VPCs. This indicates that although infants did not fully process the properties of the asynchronous face, they still showed signs of recognizing it. The reason for this can be that presentation of multiple dynamic, multimodal faces was too complex for infants to fully process during familiarization. The presence of IR may guide infants’ attention away from the properties related to face recognition. The familiarization procedure used in Experiment 1 were more complex (i.e., two dynamic faces presented simultaneously) than in previous studies. It is possible that this led the infants to be more distracted and shifted their attention away from the modality-specific features of the faces.

In Experiment 2, we familiarized infants with one dynamic face (i.e., presented with or without IR) to further explore whether 12-month-olds are able to fully process and recognize the faces in multimodal settings. Single presentations of faces may reduce the cognitive load of the task and facilitate the processing of modality-specific facial information. Infants in the synchronous condition (i.e., face presented with IR) looked longer to the face during familiarization compared to the infants in the asynchronous condition (i.e., face presented without IR), reinforcing that the idea that the presence of IR attracts infants’ attention. We found that only infants in the asynchronous condition displayed a novelty preference when the face they were familiarized with was paired with a novel face.

Our results support the intersensory redundancy hypothesis, which predicts that the presence of IR recruits attention and that the synchronous stimulation leads to earlier perception and processing of amodal properties. When IR is not present, modality-specific properties are more salient and easier to process and learn (Bahrick and Lickliter, 2000). This is likely why infants in the synchronous condition were drawn to the synchrony of the face and showed heightened attention indicated by longer looking times, but failed to move beyond the amodal information. However, because there was no synchrony in the asynchronous condition, infants were not drawn to the amodal properties and could move their attention to the modality-specific properties of the face, which enhanced recognition. Mercure et al. (2018) reported that towards the end of the first year of life, infants’ attention to the mouth region compared to the eyes of a speaking face increase. Similarly, Roth et al. (2022) found when 12-month-old infants are exposed to infant-directed speech, they focus on the mouth of a speaker. Infant-directed speech provides longer pauses, simpler sentences, and a slower rate of speech, compared to adult-directed speech and facilitates learning in infants (Thiessen et al., 2005). With these in mind, our participants were likely drawn to the mouth region of the actor speaking in an infant-directed manner, when that region provided multimodal synchrony, which in turn prevented attention to and processing of other features of the face.

This study benefitted from the use of an accessible paradigm that allowed for broad recruitment of infants across the United States, however, this approach did not provide the level of control over data collection that is seen in lab-based studies. Lookit allows researchers to collect data securely from participants from anywhere in the world and increases access of families from different backgrounds to participate in developmental research (Scott et al., 2017). Additionally, using Lookit to recruit and test participants is more time efficient compared to lab-based studies (Scott and Schulz, 2017). However, data collection and processing with Lookit presents some concerns. While our sample size is typical for lab-based infant visual attention experiments (e.g., Reynolds et al., 2013), completion of the study in the home environment may have further introduced noise in the data through environmental variability and the increased presence of distractions. In addition, the Lookit platform is still very new and only few studies using Lookit have been published thus far (e.g., Yoon and Frank, 2019; Lapidow et al., 2021; Rocha and Addyman, 2022). These studies were conducted with 2-year-olds and preschool children. Although our group sizes were larger than the group sizes in these studies, older children are able to follow experimenter’s instructions and are likely to be more to comply with data collection. Additionally, the stimuli in both experiments were designed and created during the COVID-19 pandemic, which prevented us from meeting in person for stimulus creation and data collection. While this led us to have less control than we would normally have, it allowed us to develop skills in collecting data online.

Future work in this area may benefit from the inclusion of neural methods. These methods could provide answers about lingering questions about real-time cognitive processes. Methods such as event-related potentials (ERPs) can provide additional insight into face processing and attention allocation. Examination of neural responses and infant looking behavior would allow for relations between cognitive processes and overt behavior to be better understood (Reynolds and Guy, 2012). Past studies have shown that when used together, ERPs can provide insight into recognition patterns that are not apparent from analysis of looking behavior alone (e.g., Nelson and Collins, 1992; de Haan and Nelson, 1997; Reynolds et al., 2010).

This study examined 12-month-olds’ face processing in synchronous and asynchronous audiovisual contexts and provided support for the intersensory redundancy hypothesis. Overall, the results from the current study indicate that when infants are presented with side-by-side audiovisual videos, it is difficult for them to move beyond the amodal stimulus properties that are highlighted through intersensory redundancy to the modality-specific properties of the faces. The exposure during familiarization was not sufficient for face recognition when infants were familiarized with two faces, which is likely because the stimuli were too complex to be processed during the familiarization period or that the multimodal stimulus presentation attracted infants’ attention to other stimulus properties and away from the features relevant to face processing. However, when they are familiarized with one audiovisual dynamic face, 12-month-olds can process an asynchronous face and recognize it, as indicated by a novelty preference. This suggests that modality-specific properties of a face are more salient in asynchronous conditions, when attention is not directed at amodal properties. This line of research meaningfully extends the literature on intersensory redundancy in infancy and the investigation of face processing in multimodal contexts.

## Data availability statement

The raw data supporting the conclusions of this article will be made available by the authors, without undue reservation.

## Ethics statement

The studies involving human participants were reviewed and approved by Loyola University Chicago Institutional Review Board. Written informed consent from the participants’ legal guardian/next of kin was not required to participate in this study in accordance with the national legislation and the institutional requirements. Written informed consent was obtained from the minor(s)’ legal guardian/next of kin for the publication of any potentially identifiable images or data included in this article.

## Author contributions

AB and AM collected the data. AB and MG wrote the manuscript. AB performed the data analyses. MG contributed to data analyses. All authors contributed to conception and design of the study, read, and approved the submitted version.

## Funding

This study was supported by Loyola University Chicago.

## Conflict of interest

The authors declare that the research was conducted in the absence of any commercial or financial relationships that could be construed as a potential conflict of interest.

## Publisher’s note

All claims expressed in this article are solely those of the authors and do not necessarily represent those of their affiliated organizations, or those of the publisher, the editors and the reviewers. Any product that may be evaluated in this article, or claim that may be made by its manufacturer, is not guaranteed or endorsed by the publisher.
